# Targeting MYC-driven lymphoma: lessons learned and future directions

**DOI:** 10.20517/cdr.2022.127

**Published:** 2023-04-12

**Authors:** Sandra Martínez-Martín, Marie-Eve Beaulieu, Laura Soucek

**Affiliations:** ^1^Peptomyc S.L., Vall d’Hebron Barcelona Hospital Campus, Barcelona 08035, Spain.; ^2^Preclinical & Translational Research Program, Vall d’Hebron Institute of Oncology (VHIO), Vall d’Hebron Barcelona Hospital Campus, Barcelona 08035, Spain.; ^3^Department of Biochemistry and Molecular Biology, Universitat Autònoma de Barcelona, Bellaterra 08193, Spain.; ^4^Institució Catalana de Recerca i Estudis Avançats (ICREA), Barcelona 08010, Spain.

**Keywords:** High-grade B-cell lymphoma, double expressor lymphoma, MYC, MYC therapies

## Abstract

MYC plays a central role in tumorigenesis by orchestrating cell proliferation, growth and survival, among other transformation mechanisms. In particular, MYC has often been associated with lymphomagenesis. In fact, MYC overexpressing lymphomas such as high-grade B-cell lymphoma (HGBL) and double expressor diffuse large B-cell lymphomas (DLBCL), are considered addicted to MYC. In such a context, MYC targeting therapies are of special interest, as MYC withdrawal is expected to result in tumor regression. However, whether high MYC levels are always predictive of increased sensitivity to these approaches is not clear yet. Even though no MYC inhibitor has received regulatory approval to date, substantial efforts have been made to investigate avenues to render MYC a druggable target. Here, we summarize the different classes of molecules currently under development, which mostly target MYC indirectly in aggressive B-cell lymphomas, paying special attention to subtypes with MYC/BCL2 or BCL6 translocations or overexpression.

## INTRODUCTION

### High-grade B-cell lymphomas

Lymphomas are very heterogeneous neoplasms that originate from the clonal expansion of B cells, T cells or natural killer (NK) cells^[1]^. They are divided into Hodgkin (HL), which represents 10% of the cases, and Non-Hodgkin (NHL), accounting for the remaining 90%^[2]^. In 2020, over half a million new cases of NHL were estimated globally^[3]^. NHLs are further subdivided according to their cell lineage, maturity of the cells and aggressiveness, following an evidence-based classification. Elaborated by the World Health Organization (WHO), such a classification has served as a global reference for the diagnosis of lymphoid neoplasms since its third edition in 2001 up to the current fifth edition published in 2022 (WHO-HAEM5)^[4]^. Additionally, the International Consensus Classification (ICC) recently published a report suggesting that the algorithm for the diagnosis of aggressive B-cell lymphomas, based on the current combination of morphology, immunophenotype, Epstein-Barr encoding region (EBER) *in situ* hybridization, fluorescent *in situ* hybridization (FISH) and B-cell clonality analysis, should be replaced by molecular genetic classification, based on mutational profile, somatic copy number alterations and structural variants, able to distinguish seven genetic subtypes with apparent clinical relevance^[5]^.

The most common aggressive NHLs in Western countries are diffuse large B-cell lymphoma (DLBCL), Mantle Cell lymphoma (MCL) and Burkitt lymphoma, which account for 31, 6 and 2% of adult cases, respectively^[6]^. Owing to their unique genomic features, biological behavior and poor clinical prognosis, the subtypes of DLBCL, formerly known as double-hit (DHL) and triple-hit (THL) lymphomas, were classified in 2016 as a new category, termed “high-grade B-cell lymphoma” (HGBL), with translocations involving *MYC* and *B-Cell Lymphoma* 2 (*BCL*2) and/or *B-Cell Lymphoma* 6 (*BCL6*). In WHO-HAEM5, tumors with *MYC* and *BCL*2 rearrangements are named DLBCL/HGBL *MYC*/*BCL*2, while HGBL-DH-*BCL*6 represents a separate entity. HGBL-DH-*BCL*6 are biologically less distinctive, hence considered genetic subtypes of either DLBCL, not otherwise specified (NOS) or HGBL, NOS. Complementarily, ICC considers double-hit (DH)-HGBL to comprise two entities: HGBL with *MYC* and *BCL*2 rearrangements, with or without *BCL*6 rearrangement (HGBL-DH-*BCL*2), and a provisional entity, HGBL-DH-*BCL*6, with *MYC* and *BCL*6 rearrangements^[7]^. In terms of mutational and gene expression profiles, DLBCL/HGBL *MYC*/*BCL*2 exhibit a mutational signature closer to that of follicular lymphoma (FL), including *Cyclic adenosine monophosphate Response Element Binding Protein* (*CREBBP*), *BCL*2, *Lysine Methyltransferase* 2*D* (*KMT*2*D*)*, MYC, Enhancer of Zeste Homolog* 2 (*EZH*2) and *Forkhead box protein O*1(*FOXO*1), while showing a gene expression profile similar to centroblasts of the germinal center (GC) dark zone. In contrast, HGBL-DH-*BCL*6 less frequently shows a Germinal center B cell-like (GCB) immunophenotype, is cytogenetically less complex and exhibits impairment of *E*2*F* targets, but not of the *TP*53 and *MYC* signaling pathway, characteristic of DLBCL/HGBL *MYC*/*BCL*2^[5,7]^.

DLBCL co-expressing MYC and BCL2, also known as double expressor lymphomas (DELs), are associated with shorter overall survival (OS) and Progression-Free Survival (PFS). Unlike DHL, whose cell-of-origin (COO) is primarily GCB, DELs are typically activated B cell-like (ABC)^[8]^. Further epidemiologic analysis of DHL/THL and DEL cases reveals an incidence of 2%-12% and 19%-34% among the DLBCL, respectively^[9]^. Given their dismal outcome, in the last few years since the WHO revised the classification, several reviews in the literature have pinpointed these diseases as an unmet medical need^[8,10-13]^.

In terms of therapeutic opportunities, DLBCL follow standard rituximab, cyclophosphamide, hydroxydaunorubicin, vincristine and prednisone (R-CHOP) therapy. Although there is no widely accepted standard approach to manage HGBL with *MYC* and *BCL*2/*BCL*6 rearrangements, retrospective comparisons show that DHL cases do better when treated with dose-intensive approaches compared to R-CHOP, like dose-adjusted etoposide, prednisone, vincristine, cyclophosphamide, hydroxydaunorubicin, and rituximab (DA-EPOCH-R), or cyclophosphamide, doxorubicin, vincristine, high-dose methotrexate/ifosfamide, etoposide, and high-dose cytarabine (R-CODOX-M/R-IVAC). Others have proposed combining it with targeted agents against MYC or BCL2, like lenalidomide, an immunomodulatory agent, which in some contexts causes MYC downregulation, or venetoclax (also known as ABT-199), a highly selective inhibitor of BCL2, as promising new approaches^[14]^.

### Burkitt lymphoma

Burkitt lymphoma (BL) is the most common NHL in children and young adults (representing 40%-50% of pediatric NHL)^[15]^. Its definition has remained unchanged both in WHO-HAEM5 and ICC. It is a very aggressive tumor, characterized by *MYC* rearrangement with immunoglobulin genes and mutations in *Transcription Factor* 3 (*TCF*3) or its negative regulator *Inhibitor of Protein Binding* 3 (*ID*3), as well as coding mutations that affect the B-cell receptor (BCR), G Protein-coupled receptor (GPCR) and Phosphatidylinositol 3-Kinase (PI3K) signaling pathways^[4]^. It is associated with a GCB phenotype and is considered highly proliferative^[7]^. However, the ICC replaced the provisional entity Burkitt-like lymphoma with 11q aberration with Large B-cell lymphoma with 11q aberration, because it is more resemblant to DLBCL than BL^[5]^.

In this context, treatment options, although different between adults and children, are mainly reduced to combinatorial chemotherapeutic regimens, like those used in other subtypes of lymphoma^[16]^.

### MYC, BCL2 and BCL6 deregulation and diagnosis of HGBL

The MYC family of oncoproteins is found to be deregulated in up to 70% of human cancers^[17]^. In physiological conditions, its expression is tightly regulated at all levels, from transcription to post-translational modifications^[18]^. Nonetheless, many of the genetic alterations present in cancer uncouple *MYC* expression from the usual regulatory constraints, either by constitutive activation of signal transduction pathways [e.g., Neurogenic locus notch homolog protein 1 (Notch), Wingless-related integration site (Wnt) and receptor tyrosine kinases (TKs)], or direct alterations of *MYC*, such as point mutations leading to protein stabilization, amplifications or translocations^[18,19]^. However, MYC overexpression is not always sufficient to drive tumorigenesis and often requires additional mutations, especially in cases where *MYC* expression induces not only proliferation, but also senescence or apoptosis^[20,21]^. Hence, these fail-safe mechanisms need to be disabled for MYC to exert its full pro-tumorigenic function.

Another family of proteins frequently mutated in cancer is BCL2, informally known as “guardians of cell death”^[22]^. These protein members display opposing functions to either induce or block the intrinsic apoptotic pathways. Physiologically, a delicate balance between pro- and anti-apoptotic proteins must be maintained^[23]^. However, resistance to apoptosis is one of the best-described cancer hallmarks. Thus, in the cancer context, it is common to observe an increase in the expression of anti-apoptotic family members, like BCL2, which happens to be one of the main contributors to B-cell lymphomagenesis^[24]^. As with MYC, BCL2 can be deregulated through various mechanisms that include (i) indirect ones, such as the activation of signaling pathways [e.g., Phosphatidylinositol 3-Kinase/Protein Kinase B (PI3K/AKT) or Nuclear Factor kappa-light-chain-enhancer of activated B cells (NF-κB)] and loss of Myeloid Leukemia 1 (MCL-1), and (ii) direct lesions, such as somatic mutations (restricted to FL with increased risk of transformation to DLBCL), amplifications, hypomethylation of the gene promoter or translocations with immunoglobulins^[25]^. Interestingly, BCL2 alone is also insufficient to induce full tumor development. Nevertheless, the cooperation of MYC and BCL2 does unleash the neoplastic transformation by simultaneously removing the brakes of cell growth and promoting cell survival^[26,27]^.

BCL6 belongs to a family of transcription factors key for the germinal center reactions. It functions by recruiting corepressors that block the transcription of over 1,000 genes involved in the proliferation and survival of healthy germinal center B-cells, including cell cycle checkpoints and DNA damage repair-related genes^[28,29]^. Notably, both MYC and BCL2 are targets of BCL6, and in normal conditions, this transrepressor downregulates both proteins^[28]^. However, there are various direct and indirect mechanisms for BCL6 deregulation. For instance, BCL6 cross-talks with proteins involved in chromatin modifications, such as EZH2, CREBBP and KMT2D. Hence, mutations in these genes can add up to an imbalanced BCL6 activity^[30]^. More directly, reduced phosphorylation leading to protein stabilization, as well as translocations or mutations on the first non-coding exon, also result in altered expression of BCL6^[30]^.

Importantly, 5%-15% of large B-cell lymphomas bear a translocation in MYC and BCL2 or BCL6, and 20%-30% of the cases are double expressors. Double expressors are defined in most studies as displaying ≥ 40% MYC positive and ≥ 50%-70% BCL2 positive cells. Such elevated expression of both proteins is caused by mechanisms other than gene rearrangements, such as amplifications, point mutations or oncogenic activation of signaling pathways^[31,32]^.

#### Diagnosis of DHL/THL and DEL

MYC/BCL2 DHL is described as the most common type of DHL (at least 65% of the diagnosed cases), followed by MYC/BCL2/BCL6 THL, being MYC/BCL6 DHL the rarest^[33,34]^. The technique of choice to detect rearrangements in MYC(8q24), BCL2(18q21) and/or BCL6(3q27) is Fluorescent In situ Hybridization (FISH). Remarkably, even though most of the MYC/BCL2 cases show overexpression of both proteins, as many as 20% of these patients lack such correlation, with lower MYC levels typically associated with better outcomes. This is also true for the other DHLs and THLs, in which the genomic translocations generally result in high levels of the respective proteins and a worse prognosis^[32]^.

Accurate diagnosis would appear to require the screening of all DLBCL patients by FISH^[35]^. While this has been agreed to be the best practice, as referred to in the WHO classification, the technique is not always routinely available. In Europe, only 40% of the countries have this capability, while another 40% rely only on referral hospitals^[36]^.

It is important to distinguish DHL from DEL, which is far more common and is also related to poor prognosis. Of note, DELs are usually determined using immunohistochemistry (IHC), a less robust technique than FISH and, thus, more susceptible to variability. Besides, different cut-offs are defined between studies, although most accepted values are 40% positive cells for MYC, 50%-70% for BCL2 and 60% for BCL6^[36,37]^. Nowakowski and Czuczman raised the question of whether successful treatments for DHL would equally succeed for DEL, given the inherent differences between their COOs^[37]^. Interestingly, Ennishi *et al.* discovered a 104-gene double-hit signature (DHITsig) able to distinguish a subpopulation of patients likely to respond to R-CHOP and identify tumors with potential targetable vulnerabilities. DHITsig-positive tumors, as expected, show more frequent MYC and BCL2 alterations than DHITsig-negative tumors, even though only one-half of the cases harbor MYC/BCL2 rearrangements^[38]^. Additionally, Sha *et al.*^[39]^ described a molecular high-grade (MHG) subgroup of DLBCL patients from a clinical trial investigating the addition of bortezomib to standard R-CHOP therapy. Such a subgroup encompassed most patients with DHL, extending the molecular identification to more than double the size of this poor-prognosis group. The authors suggest that MHG patients could benefit from intensified chemotherapy or novel targeted therapies^[39]^. Interestingly, in an independent study from the same authors, Cucco *et al.* identify a novel association between MHG-DLBCL with *MYC* hotspot mutations that lead to its stabilization and enhance its transforming capacity^[40]^. Studies like these emphasize the relevance of expanding our knowledge of the molecular mechanisms underlying the disease to best adequate the treatment.

Given the well-established role of MYC protein in driving lymphoma progression and the numerous preclinical studies in which suppression of MYC activity triggered regression of many tumor types considered MYC-driven^[41-44]^ - including those where MYC is not deemed the initiating oncogenic lesion^[45,46]^, it is clear that MYC inhibition could represent an effective treatment avenue.

In this review, we summarize the active preclinical and early-phase clinical research exploring novel approaches for the treatment of HGBL, focusing on MYC targeting therapies.

### MYC-targeted therapies

In the last few years, several novel approaches have been explored to improve the poor outcome observed in lymphoma patients with the subtypes of DHL or THL, as well as DEL, even though the latter is not considered an independent entity. Many of the targeted therapies proposed for these specific tumors target MYC only indirectly, causing its downregulation in most cases. Here we list the different compounds classified by their primary mechanism of action [Table 1] and refer to the preclinical and clinical data available for each of them.

**Table 1:** Summary of the indirect MYC-targeting approaches and their development stage in high-grade B-cell lymphoma

**Compound**	**Combined with other therapies?**	**Mechanism of action**	**Clinical or Preclinical**	**References**
CC-95775	No	Reduction of MYC translation via inhibition of the BET family of proteins (BRD4, BRD3, BRD2 and BRDT)	Phase Ib Completed (NCT04089527)	NA
GSK525762C	+ entinostat		Phase I Withdrawn - Protocol moved to disapproved (NCT03925428)	NA
RO6870810	+ venetoclax with or without rituximab		Phase Ib Completed (NCT03255096)	Dickinson *et al.*^[54]^
Chidamide	+ rituximab	HDAC inhibitors	Preclinical	Guan *et al.*^[57]^
	+ high-dose rituximab and chemotherapy followed by auto-HSCT		Clinical - Case Report	Kang *et al.*^[58]^
	+ venetoclax		Preclinical	Luo *et al.*^[59]^
Marbostat-100	No		Preclinical	Winkler *et al.*^[55]^
CKD-581	No		Preclinical	Kim *et al.*^[61]^
			Phase I Completed (NCT01580371)	NA
CUDC-101	+ gemcitabine		Preclinical	Li *et al.*^[62]^
Compound 8	No		Preclinical	Zhang *et al.*^[63]^
Tucidinostat	+ R-CHOP		Phase III Recruiting (NCT04231448)	Zhang *et al.*^[64]^
Fimepinostat (CUDC-907)	No	Dual HDAC and PI3K inhibitor	Phase II Completed (NCT02674750)	Landsburg *et al.*^[65]^
VIP152	+ immunotherapy	CDK9 inhibitors	Phase I Recruiting (NCT02635672)	Diamond *et al.*^[67]^
BTX-A51	No		Phase I Recruiting (NCT04872166)	Ball *et al.*^[70]^
KB-0742	No		Phase I Recruiting (NCT04718675)	NA
SHC014748M	No	PI3K inhibitors	Phase I Unknown (NCT03588598)	Fan *et al.*^[80]^
WNY1613	No		Preclinical	Zuo *et al.*^[81]^
CYH33	No		Preclinical	Chen *et al.*^[82]^
SAF-248	No		Preclinical	Zhang *et al.*^[83]^
KA2237	No		Phase I Completed (NCT02679196)	Nastoupil *et al.*^[84]^
BR101801	No		Phase I/II Recruiting (NCT04018248)	Kim *et al.*^[86]^
Parsaclisib	+ R-CHOP		Phase I/Ib Recruiting (NCT04323956)	Wang *et al.*^[88]^
TG-1701	No		Preclinical	Ribeiro *et al.*^[90]^
Ibrutinib	+ CIT (chemoimmunotherapy)		Phase III Terminated (NCT02703272)	Burke *et al.*^[91,92]^
Zanubrutinib	+ R-CHOP	BTK inhibitors	Phase II Recruiting (NCT05189197)	NA
Acalabrutinib	+ CAR T cell		Phase II Not yet recruiting (NCT05583149)	NA
	+ ACP-319 (PI3Kδi)		Phase I/II Active, not recruiting (NCT02328014)	Barr *et al.*^[94]^
Pimozide	+ etoposide		Preclinical	Li *et al.*^[100]^
CS2164 (Chiauranib)	+ venetoclax		Preclinical	Yuan *et al.*^[103]^
eFT226	+ AKTi/PI3Ki	USP1 inhibitor	Preclinical	Thompson *et al.*^[104]^
Ixazomib	+ DA-EPOCH-R	Multitarget inhibitor (VEGFR1, VEGFR2, VEGFR3, and c-Kit, CSF-1R, AURKB)	Phase I/II Active, not recruiting (NCT02481310)	Galvez *et al.*^[108]^
Bortezomib	+ R-CHOP	eIF4A inhibitor	Phase III Completed (NCT01324596)	Davies *et al.*^[109,110]^
Alisertib	+ romidepsin	Proteasome inhibitors	Phase I Completed (NCT01897012)	Strati *et al.*^[111]^
MRT-2359	No		Phase I/II Recruiting (NCT05546268)	Gavory *et al.*^[112]^
		Aurora A kinase inhibitor		
		Degradation of GSTP1 and downregulation of N- and L-MYC		

BTK: Bruton’s tyrosine kinase; BET: bromodomain extra-terminal; DA-EPOCH-R: dose-adjusted etoposide, prednisone, vincristine, cyclophosphamide, hydroxydaunorubicin, and rituximab; HDAC: Histone deacetylase; R-CHOP: rituximab, cyclophosphamide, hydroxydaunorubicin, vincristine and prednisone; USP1: ubiquitin-specific protease 1; VEGFR: vascular endothelial growth factor receptor.

#### Bromodomain extra-terminal inhibitors

Bromodomain extra-terminal (BET) inhibitors have been a promising class of drugs in the cancer field for the past 10 years^[47]^. Inhibition of these transcriptional regulators can result in the silencing of MYC expression when it is under the control of super-enhancer elements^[48]^. A first-generation BETi, JQ1, for instance, was used in DHL/THL cells and showed the ability to slow down cell growth and induce apoptosis in a dose-dependent manner, and increased the therapeutic effect of the BCL2 inhibitor venetoclax^[49,50]^. A similar combination was later tested with another BETi, CPI203, which achieved simultaneous downregulation of MYC and Bcl-2-related protein A1 (BFL-1), overcoming the emergence of resistance to venetoclax both in DHL cultures and tumor xenografts^[51]^.

Notably, it was expected that high MYC levels would be predictive of enhanced sensitivity to BETi. However, this is not always the case. For example, independent preclinical studies of different hematologic malignancies evidenced that MYC amplification failed to predict the sensitivity to the BETi OXT015^[52]^. Moreover, in a Phase I clinical trial with prostate cancer patients, there was a lack of correlation between the reduction in MYC levels and the response to the pan-BET inhibitor Zen-3694^[52]^. Similarly, Li *et al.*^[53]^ demonstrated that several DHL/THL cell lines were as sensitive to various BETis (I-BET-762, JQ1 and OTX015) as U2932, a lymphoma cell line with no MYC rearrangement. Regarding BETi in clinical development for high-grade B-cell lymphoma or other NHLs, a couple of Phase I trials were completed (NCT04089527) or withdrawn (NCT03925428) in 2022 and 2020, respectively, but no reports have been published yet. Encouragingly, Dickinson *et al.*^[54]^ tested the BETi RO6870810 in combination with venetoclax (a BCL2 inhibitor) and rituximab (an anti-CD20 monoclonal antibody). In this Phase Ib study (NCT03255096), no DHL patients were enrolled, but 10 out of 18 patients (55.6%) were considered DEL. From those, one patient achieved a complete response (CR).

#### Histone deacetylase inhibitors

Another group of targeted agents that have been under development for even longer (over 30 years) than BETi and represented hope for the treatment of hematologic diseases includes Histone deacetylase (HDAC) inhibitors (HDACi). MYC can recruit epigenetic modifiers, like HDACs, to activate or repress different target genes. Notably, MYC is found acetylated at K423 upon treatment with a pan-HDACi, decreasing MYC transcription due to autoregulation and resulting in apoptosis^[55]^.

Overall, though, the accumulated clinical data with these therapeutics has been somewhat disappointing, as they did not fulfill the promise of the *in vitro* and *in vivo* models. Indeed, only certain hematologic tumors seem to benefit from this type of treatment in the clinical setting, with only five approved drugs, of which four are for T-cell lymphoma and one for multiple myeloma. Interestingly, breast cancer was the first indication, outside hematologic malignancies, to get the approval of an HDAC inhibitor as a combinatorial therapy (tucidinostat plus exemestane, an aromatase inhibitor) in 2019^[56]^.

In the particular case of HGBL, several compounds are under investigation. At the preclinical level, Guan *et al.*^[57]^ identified the promising combination of chidamide (an HDACi approved by the China Food and Drug Administration for T-cell lymphoma) and rituximab (a monoclonal antibody against CD20) as a potential strategy to treat relapsed/refractory (R/R) DLBCL. Indeed, such a combination led to an impressive reduction of tumor volume in a DLBCL cell line-derived xenograft mouse model. The same authors also report the clinical case of a patient that achieved partial response (PR) after only one cycle and underwent CR after three cycles of treatment^[57]^. Similarly, Kang *et al.*^[58]^ studied the effect of a modified conditioning regimen with chidamide and high-dose rituximab for THL, followed by autologous hematopoietic stem cell transplantation (auto-HSCT), showing 2 CRs, while a third patient, who was insensitive to the chemotherapy, did not respond.

In a different study, Luo *et al.*^[59]^ proposed the combination of chidamide with venetoclax for the treatment of DHL. They demonstrated a synergistic effect of the compounds both *in vitro* and *in vivo* using SU-DHL-4 (MYC/BCL2 DEL) and DB (*MYC*/*BCL*2 rearranged) cell lines. Importantly, chidamide caused a reduction in MYC levels in both cell lines, suggesting that suppression of MYC is achieved regardless of the rearrangement status^[59]^.

In contrast, the research by Winkler *et al.*^[55]^ described a new molecule, Marbostat-100 (M-100), an HDAC6 inhibitor that targets almost exclusively lymphoma cells with high MYC levels. Contrary to previous observations with the pan-HDACi MS-275, M-100 caused MYC reduction by proteasomal degradation, while *MYC* transcription was not affected, and extended survival of transgenic Eμ-*myc* mice *in vivo*. Importantly, the authors underline that MYC needs to be Threonine58 (T58) wild type for its degradation to happen efficiently^[55]^, as a mutation of this residue on the MYC protein prevents a phosphorylation event critical to its proficient proteasomal degradation^[60]^. In fact, Cucco *et al.*^[40]^ identified a high frequency of *MYC* T58A and P57S mutations in the MHG subset of DLBCL.

Kim *et al.*^[61]^ assessed the efficacy of CKD-581, another HDACi that was previously tested in a Phase I clinical trial (NCT01580371), but for which no safety and pharmacokinetic results were published. Preclinically, this broad-spectrum HDACi decreased the expression of both MYC and BCL2 and was able to elicit a significant reduction in tumor growth. Similarly, other recent preclinical studies showed the potential of HDACi for the treatment of some types of NHL^[62,63]^.

More advanced in their development, tucidinostat and fimepinostat (CUDC-907) have been or are being evaluated in Phase II/III trials (NCT02909777, NCT02674750). Zhang *et al.*^[64]^ showed improved 2-year progression-free survival (PFS) and overall survival (OS) in DEL patients, as well as a lessened negative prognostic impact of *CREBBP*/*EP*300 (*Histone acetyltransferase p*300) mutations when treated with tucidinostat in combination with R-CHOP. Furthermore, fimepinostat is a dual HDAC and PI3K inhibitor reported to have an objective response rate (ORR) of 15% in patients with relapsed/refractory DLBCL or HGBL with high MYC (≥ 40%). ORR was further increased to 22% when the patients were classified based on a 3-protein biomarker. Such a discovery led the authors to suggest that combinatorial therapies or biomarker-assisted stratification could improve even more the response to treatment^[65]^.

#### Cyclin-dependent kinase 7/9 inhibitors

Cyclin-dependent kinase (CDK) inhibitors have been the topic of intense research for two decades. In particular, CDK9 emerged as a druggable target for the development of cancer therapeutics, due to its crucial role in the transcriptional regulation of both short-lived anti-apoptotic proteins and oncogenes, such as *BCL*2/6 and *MYC*, critical for the survival of transformed cells^[66]^.

VIP152 is a potent and highly selective CDK9 inhibitor that, in a Phase I study, led to complete metabolic remission by PET-CT in two out of seven patients with HGBL^[67]^, and stabilization of the disease in seven out of 30 patients with non-lymphoma solid tumors^[67,68]^. Preclinical models using the SU-DHL-10 HGBL cell line in a murine xenograft recapitulated the remission observed clinically in a dose-dependent manner. Additionally, pharmacodynamic biomarker analysis *in vitro* demonstrated a transient downregulation of the mRNA of MYC, MCL-1 and Proliferating Cell Nuclear Antigen (PCNA), resulting in a durable clearance of these oncogenic drivers^[69]^. Frigault *et al.*^[69]^ also reported a transient biomarker modulation in 7 stable HGBL patients of their study when evaluating short-lived transcripts by RNAseq from whole blood samples. In particular, they observed a 70% reduction in MYC, MCL1 and PCNA mRNA post-dose.

Ball *et al.*^[70]^ presented at the 2022 ASCO annual meeting the interim results of the first-in-human trial of BTX-A51 (NCT04872166), a direct inhibitor of casein kinase 1α (CK1α), CDK7 and CDK9 that robustly increases p53 protein levels via CK1α inhibition, while preferentially decreasing super-enhancer transcription of MYC and MCL-1. In the second part of the study, they are planning to enroll up to 40 additional subjects to evaluate the safety and preliminary efficacy in patients with documented MYC genomically amplified or overexpressed tumors, such as HGBL (NHL)^[71]^. BTX-A51 in monotherapy has been found to have an acceptable safety profile and promising antileukemic activity^[71]^.

Another interesting orally bioavailable CDK9i is KB-0742, which demonstrated preclinical efficacy in AR-dependent castration-resistant prostate cancer^[72]^ and transcriptionally addicted tumors, such as sarcoma and chordoma^[73]^. It is currently being tested in a Phase I clinical trial that includes DLBCL with MYC translocation and Burkitt lymphoma (NCT04718675).

#### Phosphoinositide 3-kinases inhibitors

PI3K/AKT/mTOR (PAM) signaling is involved in important physiological and pathophysiological functions that drive tumor progression, such as metabolism, cell growth, proliferation, angiogenesis and metastasis^[74]^. In particular, different lymphoma subtypes (a subset of DLBCL and BL) have been shown to rely on BCR survival signals mediated by the phosphoinositide 3-kinases (PI3K) pathway to promote the proliferation and survival of malignant B-cells^[75-77]^. One of the main downstream targets of the PAM pathway is MYC: phosphorylation at T58 occurs via glycogen synthase kinase 3 (GSK3B), which is a direct target of AKT^[78]^. Moreover, activation of mTOR and AKT activity directly increases the translation of MYC^[78]^ and activation of PAM leads to the phosphorylation and degradation of Mad1, the natural antagonist of MYC^[79]^. Pharmacological suppression of this pathway has been proposed as an anti-neoplastic approach. Small-molecule inhibitors targeting PI3K consist of pan-, isoform-specific and dual PI3K/mTOR (Mammalian Target of Rapamycin) inhibitors.

Several groups in China have identified new PI3Ki and tested them against panels of B-cell lymphoma cell lines, including DLBCL and some examples of HGBL, such as DOHH2, SU-DHL-6 and SU-DHL-10:

Fan *et al.*^[80]^ showed *in vitro* activity of their compound SHC014748M, a PI3Kδ inhibitor, which also caused a significant reduction in tumor volume in a xenograft mouse model with the SU-DHL-6 cell line. Remarkably, SHC014748M performed better than idelalisib, an FDA-approved drug for the treatment of NHL. Currently, SHC014748M is being evaluated against indolent relapsed/refractory B-cell NHL, including chronic lymphocytic leukemia (CLL), FL, marginal zone lymphoma (MZL) and Waldenstrom’s macroglobulinemia (WM), in a Phase I clinical trial (NCT03588598).

Similarly, Zuo *et al.*^[81]^ found their candidate WNY1613, also a PI3Kδ inhibitor, to exert antiproliferative effects *in vitro* in a panel of NHL cell lines (seven DLBCL, two mantle cell lymphoma (MCL) and one BL). They also demonstrated that their inhibitor can prevent tumor growth in two mouse xenograft models of SU-DHL-6 and JEKO-1 cell lines. The pharmacodynamics and pharmacokinetics of this molecule are under current investigation.

In independent studies, Chen *et al.*^[82]^ (using CYH33 against PI3Kα) and Zhang *et al.*^[83]^ (using SAF-248 against PI3Kδ) evidenced that both compounds were effective in blocking B-cell lymphoma cell growth *in vitro* and *in vivo*, partly by causing the downregulation of MYC. As happened with SHC014748M, SAF-248 displayed superior activity compared to idelalisib, which is also reported to be far more toxic. Importantly, Zhang *et al.* demonstrated that the expression of PI3Kα was negatively correlated with the activity of SAF-248 and found multiple oncogenic pathways, such as IL2_STAT5 (Interleukin-2/Signal Transducer and Activator of Transcription 5), IL6_STAT3 (Interleukin-6/Signal Transducer and Activator of Transcription 3), MTORC1 (Mammalian Target Of Rapamycin Complex 1) and MYC, upregulated in tumor tissues upon prolonged treatment with SAF-248 compared to those with short-term administration, possibly pinpointing to an adaptive mechanism of resistance^[83]^. In line with this observation, Chen *et al.*^[82]^ proposed the combination with BETi or HDACi to overcome the adaptive resistance to PI3K inhibition, which could be attributed to increased H3K27Ac and binding of CREB Binding Protein (CBP)/p300 with BRD4 proteins gene loci of a subset of growth factors and receptors.

Some other PI3Ki under clinical development for the treatment of B-cell lymphoma include KA2237, BR101801 and parsaclisib. Nastoupil *et al.*^[84]^ reported KA2237, a dual PI3K β/δ inhibitor, to have a manageable toxicity profile and to be an effective therapeutic option for patients with refractory B-cell lymphoma without an acceptable standard of care option. In a Phase I clinical study with the compound, 19 out of 21 patients were evaluable for response, and 8 of the evaluable ones had DLBCL. Two of the DLBCL patients achieved CRs and another one achieved a PR^[84]^.

BR101801, a triple inhibitor of PI3K γ/δ and DNA-PK, showed highly potent effects in blocking cellular proliferation of NHL cell lines in preclinical models, including both the indolent and aggressive subtypes^[85]^. Clinically, the compound was well tolerated and showed preliminary signs of activity in patients with relapsed/refractory hematologic malignancies. The Phase Ib/II study of BR101801 is warranted in relapsed/refractory NHL^[86]^.

Finally, parsaclisib, the most advanced PI3Ki in clinical development described here, inhibits PI3K δ. Shin *et al.*^[87]^ found that overexpression of MYC turned some of the DLBCL cell lines insensitive to parsaclisib treatment. The resistance could be overcome by the addition of a BETi that reduced MYC levels. Recently, Wang *et al.*^[88]^ investigated the feasibility of combining parsaclisib with standard immunochemotherapy (R-CHOP), seeking signs of efficacy. They chose the population of study to be high-risk lymphoma patients bearing MYC rearrangements or translocations (i.e., HGBL) or overexpression of MYC or BCL2. In the interim report, 13 patients were evaluable, 8 achieved CR, 4 PR and one progressed^[88]^. Given the encouraging preliminary efficacy, parsaclisib plus R-CHOP could constitute an experimental arm in future frontline DLBCL trials investigating genetic subtype-driven novel therapies.

#### Bruton’s tyrosine kinase inhibitors

Ever since the discovery of the involvement of tyrosine kinases (TK) in cancer, which led to their consideration as valuable targets for cancer treatment, a broad spectrum of TKis has been launched, including Bruton’s tyrosine kinase inhibitors (BTKis). Previous genomic studies suggested that some components of the B-cell receptor (BCR) signaling pathway are MYC transcriptional targets. Moreover, it has been demonstrated that, in pre-malignant B cells from the Eμ-*myc* mouse model, MYC overexpression is sufficient to activate BCR and PI3K/AKT signaling pathways, while conferring resistance to pharmacologic inhibitors of the BCR signaling pathway, like BTKis^[89]^.

For the treatment of B-NHL, some preclinical studies with TG-1701 showed improved efficacy compared to ibrutinib, the first BTKi approved by the FDA, and described MYC downregulation, both at the mRNA and protein level, as part of the signature observed in early-responder patients, as well as in BTKi-sensitive B-NHL cell lines and xenografts^[90]^.

In this sense, Burke *et al.*^[91]^ reported promising preliminary efficacy findings in a Phase III clinical trial with pediatric patients with R/R mature B-NHL, who have a poor prognosis, treated with ibrutinib in combination with rituximab, ifosfamide, carboplatin and etoposide (RICE) modified with dexamethasone or with rituximab, vincristine, ifosfamide, carboplatin, idarubicin and dexamethasone (RVICI). More than half of the patients (12/21) responded to the therapies, achieving a total of 5 CR and 7 PR. However, as the final endpoint was event-free survival (EFS), the study was stopped early for futility, given that ibrutinib did not improve EFS in this population in combination with chemotherapy backbones. The authors suggest prioritizing, instead, bispecific antibodies, antibody-drug conjugates and CAR-T cells^[92]^.

Even though BTKis have shown modest therapeutic activity in DLBCL (i.e., ORR of 23% for ibrutinib in relapsed patients, of 24% for acalabrutinib in DLBCL patients regardless of their molecular subtype, and of 36% for zanubrutinib in ABC DLBCL patients), Yang *et al.*^[93]^ performed retrospective biomarker assessments and showed that zanubrutinib could have antitumor activity in patients with mutations in Cluster of Differentiation 79-B (CD79B) and Myeloid Differentiation primary response 88 (MYD88). Hence, they propose future studies to focus on developing mechanism-based treatment combinations and biomarker-driven patient selection. In line with this reflection, Fudan University is evaluating zanubrutinib in combination with the immunochemotherapy R-CHOP in patients with non-GCB DLBCL with co-expression of MYC/BCL2, based on the findings of a posthoc analysis on four studies, in which an ORR of 61% and PFS of 5.4 months was achieved in patients with MYC and BCL2 overexpression treated with zanubrutinib (NCT05189197).

Acalabrutinib, as zanubrutinib, is another potent selective, irreversible BTKi with minimal off-target effects, being evaluated in combination with CAR-T cell therapy or with PI3Ki in Phase II clinical trials (NCT04257578). Barr *et al.*^[94]^ investigated the combination of acalabrutinib with ACP-319 in relapsed/refractory B-cell NHL. They showed in patients with non-GCB DLBCL an ORR of 63% (10 out of 16 patients; CR rate 25%) with a median duration of response of 8.2 months. Of the ten responders, six were double expressors, overexpressing MYC and BCL2/BCL6. No responses were observed in the nine patients with GCB DLBCL. Another Phase II study should start soon to evaluate the effectiveness and safety of acalabrutinib combined with lisocabtagene maraleucel (liso-cel) in relapsed/refractory aggressive B-cell lymphoma patients (NCT05583149).

#### MYC/MAX antagonists

While all the approaches listed above have targeted MYC indirectly, there is also the possibility of attacking it directly. Perhaps the most common strategy in this context focuses on the disruption of the interaction of MYC with its natural partner MAX. In this case, most data in the literature are related to the use of small molecules (SM), although MYC lacks significant secondary and tertiary structure when not complexed with other proteins, making specific recognition by SM quite difficult^[95]^. The first one to show effect in lymphoma was 10058-F4, which was employed *in vitro* in different BL cell lines and demonstrated that targeting of MYC/MAX interaction could impair lymphoma growth in a time- and dose-dependent manner^[96]^. Later on, 7-nitro-*N*-(2-phenylphenyl)-2,1,3-benzoxadiazol-4-amine (10074-G5) was used in Daudi BL cells, where, again, it inhibited cell growth *in vitro*, but failed to affect growth in xenografts in C.B-17 SCID mice, likely due to poor bioavailability^[97]^. Another SM, sAJM589, was more recently identified in a Principal Component Analysis (PCA)-based high-throughput screen. sAJM589 was able to inhibit the transcription of MYC target genes in P493-6 BL cells, as well as to suppress the proliferation of diverse MYC-dependent cancer cell lines and anchorage-independent growth of Raji cells^[98]^. However, the most advanced MYC/MAX dimerization inhibitor in clinical development is Omomyc, a MYC dominant negative based on the basic helix-loop-helix leucine zipper (b-HLH-Z) domain of the human c-MYC protein, able to form homodimers and heterodimers with MYC and MAX and interfere with MYC transcriptional activity. Omomyc has been used in its transgenic form in BL, in murine lymphoma cell lines obtained from Eμ*-myc* transgenic mice and in Raji cells (wild-type or knock out for FBXO11) *in vitro* and *in vivo* (subcutaneous xenografts), alone or in combination with the BCL6 degrader BI-3802^[99]^. In this context, Omomyc blocked cell proliferation and increased apoptosis, effects further improved by combined BCL6 targeting. Remarkably, the first Omomyc-derived drug product, OMO-103, has just successfully completed a Phase I clinical trial (NCT04808362) in all-comers solid tumors. However, its safety and efficacy in hematologic diseases have not been tested in the clinical setting yet. Hopefully, this will soon be the case.

#### Other inhibitors and degraders

Further preclinical or clinical studies are assessing the efficacy of novel targets or repurposed molecules for the treatment of MYC-overexpressing lymphoma.

Li *et al.*^[100]^ elucidated the role of ubiquitin-specific protease 1 (USP1) in B-cell lymphoma. USP1 is highly expressed in DLBCL patients and was found to be associated with poorer prognosis. Using pimozide, a USP1 inhibitor, the authors could stop DLBCL cells from cycling. Mechanistically, they found USP1 to stabilize MAX, promoting the transcription of MYC target genes. Importantly, when combined with etoposide, a chemotherapeutic, pimozide blocked the tumor growth in a xenograft mouse model resistant to immunochemotherapy R-CHOP. Since pimozide is already approved by the FDA for the treatment of other diseases and is known to cause low toxicity, the authors propose the combination as an attractive alternative for DLBCL patients resistant to R-CHOP.

On a different note, CS2164 is an orally bioavailable multitargeted inhibitor being evaluated in numerous clinical studies with promising clinical anti-cancer effects and tolerable toxicities^[101]^. Deng *et al.* observed a potential role of CS2164 for the treatment of NHLs through the perturbation of multiple signaling cascades (angiogenesis, inflammation and proliferation) by inhibiting the following kinases: vascular endothelial growth factor receptor 1-3 (VEGFR1, VEGFR2 and VEGFR3), platelet-derived growth factor receptor alpha (PDGFRα), receptor tyrosine kinase (c-Kit), kinase Aurora B (AURKB), and chronic inflammation-related kinase (CSF-1R). Specifically, CS2164 showed superior anti-lymphoma activity against MYC-arranged BL models, suggesting it could also have a cytotoxic effect on other MYC-altered malignancies^[102]^. Following these observations, Yuan *et al.*^[103]^ assessed the efficacy of this novel agent in combination with venetoclax using HGBL *in vitro* and *in vivo* models, where they could demonstrate a reduction of MYC and BCL2 protein levels, as well as antitumor efficacy with tolerable toxicities in a xenograft mouse model with MCA cells.

Interestingly, Thompson *et al.* found that activation of the PI3K/mTOR signaling pathway results in the activation of eukaryotic initiation factor-4A (eIF4A), required for the translation of oncoproteins, like MYC. The authors used eFT226 to inhibit the translation of specific mRNAs by promoting eIF4A1 binding to 5′-untranslated regions (UTR) containing polypurine and/or G-quadruplex recognition motifs^[104]^. The compound blocked the proliferation of GCB-DLBCL tumor models Pfeiffer and SU-DHL6. When assessing the pharmacodynamic response to the drug, the authors reported downregulation of MYC, Cyclin D1, and BCL6 protein levels in a time- and exposure-dependent manner. Collectively, their results support the clinical development of eFT226 in patients with B-cell malignancies.

A different class of molecules that emerged as an important therapeutic strategy in hematologic malignancies, particularly in multiple myeloma, are proteasome inhibitors^[105]^. Ravi *et al.*^[106]^ demonstrated that ixazomib sensitivity is mediated through checkpoint kinase 1 (CHK1)-dependent MYC function involving histone acetylation, and demonstrated that dual inhibition of CHK1 and MYC induced synergistic cell death with ixazomib. Based on preclinical data showing MYC downregulation, a couple of clinical studies were started to evaluate the efficacy of ixazomib in lymphoma patients with different subtypes (DLBCL, FL, MZL, MCL, transformed FL, DLBCL-FL, and some other indolent forms) in combination with immune- or immunochemotherapy. Graf *et al.*^[107]^ concluded that once-weekly oral ixazomib showed a favorable safety profile and considerable activity in the frontline treatment of indolent B-cell NHL, with the best results in FL. When combined with a single 4-week course of rituximab, ixazomib achieved durable disease control with very low toxicity in the majority of patients with FL. On the other hand, Galvez *et al.* reported DA-EPOCH-R induction with adjunctive ixazomib followed by maintenance ixazomib to be safe and effective in an older population of HGBL. The ORR after induction was 89%, with an associated CR rate of 61%^[108]^.

Moreover, Sha *et al.*^[39]^ reported that the addition of bortezomib to the R-CHOP regimen (RB-CHOP) could be beneficial for the MHG subgroup in a randomized Phase III study (NCT01324596). Indeed, Davies *et al.* presented at the ASH 2022 Annual Meeting extended data on the REMoDL-B trial, where 5-year PFS was significantly improved for MHG patients (PFS 29% with R-CHOP *vs.* 55% with RB-CHOP)^[109,110]^.

Lastly, Strati *et al.*^[111]^ recently (2020) discussed the results of their Phase I trial assessing the combination of alisertib (an Aurora A kinase inhibitor) with romidepsin (and HDACi) for the treatment of patients with relapsed/refractory B- and T-cell lymphomas (NCT01897012). Unfortunately, the cytokinesis failure observed in preclinical models when combining both agents only happened in T-cells in the clinical setting. Hence, significant myelosuppression and limited ORR (< 20%) for B-cell NHL patients do not support further clinical evaluation of alisertib.

On a more positive note, novel promising agents, such as MRT-2359, a molecular glue degrader directed against GSTP1 developed by Monte Rosa Therapeutics, are under development. Preclinical results on MRT-2359 were presented last year at the AACR Annual Meeting and the 34^th^ EORTC-NCI-AACR Symposium, with the promise of being effective in MYC-driven tumors addicted to protein translation^[112]^. MRT-2359 is being evaluated in a Phase I/II trial that started recruitment very recently (NCT05546268)

## CONCLUSION

Assessment of MYC rearrangements or expression is typically used in lymphomas and other hematologic malignancies as a diagnostic tool^[113,114]^. These cancers have gained the classification of MYC-associated tumors, because this oncoprotein has been demonstrated to play a key role in the physiopathology of these diseases^[115,116]^. In fact, according to the concept of oncogene addiction, multiple studies have shown that MYC inactivation causes tumor regression in the context of lymphomas^[41-44]^. In this review, we summarized the approaches explored so far to target MYC-dependency, *in vitro*, *in vivo* and in the clinical setting. However, it is important to stress that most of these approaches have been so far indirect ones and there is no set of experiments that established to which extent different cancer cells are dependent on MYC activity or, in other words, how much MYC inhibition is required to stop their proliferation or cause cell death.

Based on the above, several questions remain still unanswered: (i) would direct MYC inhibition be a more effective therapeutic strategy than indirect targeting of it in the context of lymphoma? (ii) are MYC levels a good predictive biomarker of response to MYC inhibition in every context? (iii) what is the ideal way of assessing MYC function? Would mRNA expression, protein expression, and determination of gene amplifications or rearrangements be sufficient? (iv) is MYC activity a biomarker that can potentially stratify good and bad responders to therapies in general? (v) is it possible to define a unique MYC transcriptional signature shared across various tumor types?

Our knowledge continues to expand as more efforts are dedicated to designing viable therapies that target MYC. Nevertheless, we believe it is critical answering some of the questions above to improve our understanding of MYC biology and develop the best MYC-targeting approaches.

## DECLARATIONS

### Copyright

© The Author(s) 2023.

## References

[1] Jaffe ES (2019). Diagnosis and classification of lymphoma: Impact of technical advances. Semin Hematol.

[2] Mugnaini EN, Ghosh N (2016). Lymphoma. Prim Care.

[3] https://gco.iarc.fr/today/online-analysis-pie?v=2020&mode=population&mode_population=regions&population=900&populations=900&key=total&sex=0&cancer=34&type=0&statistic=5&prevalence=0&population_group=0&ages_group%5B%5D=0&ages_group%5B%5D=17&nb_items=7&group_cancer=1&include_nmsc=1&include_nmsc_other=1&half_pie=0&donut=0.

[4] Alaggio R, Amador C, Anagnostopoulos I (2022). The 5th edition of the world health organization classification of haematolymphoid tumours: lymphoid neoplasms. Leukemia.

[5] Campo E, Jaffe ES, Cook JR (2022). The international consensus classification of mature lymphoid neoplasms: a report from the clinical advisory committee. Blood.

[6] Thandra KC, Barsouk A, Saginala K, Padala SA, Barsouk A, Rawla P (2021). Epidemiology of non-Hodgkin’s lymphoma. Med Sci.

[7] Falini B, Martelli MP (2023). Comparison of the international consensus and 5th WHO edition classifications of adult myelodysplastic syndromes and acute myeloid leukemia. Am J Hematol.

[8] Zhuang Y, Che J, Wu M (2022). Altered pathways and targeted therapy in double hit lymphoma. J Hematol Oncol.

[9] Mehta A, Verma A, Gupta G, Tripathi R, Sharma A (2020). Double hit and double expresser diffuse large B cell lymphoma subtypes: discrete subtypes and major predictors of overall survival. Indian J Hematol Blood Transfus.

[10] Khelfa Y, Lebowicz Y, Jamil MO (2017). Double-hit large B cell lymphoma. Curr Oncol Rep.

[11] Chiappella A, Crombie J, Guidetti A, Vitolo U, Armand P, Corradini P (2019). Are we ready to treat diffuse large B-cell and high-grade lymphoma according to major genetic subtypes?. HemaSphere.

[12] Azizian NG, Liu Y, Pham LV, Li Y (2019). Rational targeted therapeutics for double-hit lymphoma. Int J Hematol Oncol.

[13] Di M, Huntington SF, Olszewski AJ

[14] Dunleavy K (2021). Double-hit lymphoma: optimizing therapy. Hematology.

[15] Frazer JK, Li KJ, Galardy PJ (2019). Excellent outcomes in children and adolescents with CNS^+^ Burkitt lymphoma or other mature B-NHL using only intrathecal and systemic chemoimmunotherapy: results from FAB/LMB96 and COG ANHL01P1. Br J Haematol.

[16] Saleh K, Michot JM, Camara-Clayette V, Vassetsky Y, Ribrag V (2020). Burkitt and burkitt-like lymphomas: a systematic review. Curr Oncol Rep.

[17] Hann SR (2014). MYC cofactors: molecular switches controlling diverse biological outcomes. Cold Spring Harb Perspect Med.

[18] Dang CV (2012). MYC on the path to cancer. Cell.

[19] Schaub FX, Dhankani V, Berger AC (2018). Pan-cancer alterations of the MYC oncogene and its proximal network across the cancer genome atlas. Cell Syst.

[20] Gabay M, Li Y, Felsher DW (2014). MYC activation is a hallmark of cancer initiation and maintenance. Cold Spring Harb Perspect Med.

[21] Dhillon P, Evan G (2019). In conversation with Gerard evan. FEBS J.

[22] Daniel PT, Schulze-Osthoff K, Belka C, Güner D (2003). Guardians of cell death: the Bcl-2 family proteins. Essays Biochem.

[23] Klanova M, Klener P (2020). BCL-2 proteins in pathogenesis and therapy of B-cell non-hodgkin lymphomas. Cancers.

[24] Adams CM, Clark-Garvey S, Porcu P, Eischen CM (2018). Targeting the Bcl-2 family in B cell lymphoma. Front Oncol.

[25] Kapoor I, Bodo J, Hill BT, Hsi ED, Almasan A (2020). Targeting BCL-2 in B-cell malignancies and overcoming therapeutic resistance. Cell Death Dis.

[26] Strasser A, Harris AW, Bath ML, Cory S (1990). Novel primitive lymphoid tumours induced in transgenic mice by cooperation between myc and bcl-2. Nature.

[27] Fairlie WD, Lee EF (2021). Co-operativity between MYC and BCL-2 pro-survival proteins in cancer. Int J Mol Sci.

[28] Basso K, Dalla-Favera R (2010). BCL6: master regulator of the germinal center reaction and key oncogene in B cell lymphomagenesis. Adv Immunol.

[29] Basso K, Dalla-Favera R (2012). Roles of BCL6 in normal and transformed germinal center B cells. Immunol Rev.

[30] Yang H, Green MR (2019). Epigenetic programing of B-cell lymphoma by BCL6 and its genetic deregulation. Front. Cell Dev Biol.

[31] Swerdlow SH (2014). Diagnosis of “double hit” diffuse large B-cell lymphoma and B-cell lymphoma, unclassifiable, with features intermediate between DLBCL and Burkitt lymphoma: when and how, FISH versus IHC. Hematol Am Soc Hematol Educ Program.

[32] Riedell PA, Smith SM (2018). Double hit and double expressors in lymphoma: definition and treatment. Cancer.

[33] Cheah CY, Oki Y, Westin JR, Turturro F (2015). A clinician’s guide to double hit lymphomas. Br J Haematol.

[34] Quesada AE, Medeiros LJ, Desai PA (2017). Increased MYC copy number is an independent prognostic factor in patients with diffuse large B-cell lymphoma. Mod Pathol.

[35] Scott DW, King RL, Staiger AM (2018). High-grade B-cell lymphoma with MYC and BCL2 and/or BCL6 rearrangements with diffuse large B-cell lymphoma morphology. Blood.

[36] Aurer I, Kersten MJ, Dreyling M, Federico M (2019). PF322 approach to double hit lymphoma (DHL) diagnosis and treatment in europe - a cross-section study of EHA lymphoma working party (EHA LYG). HemaSphere.

[37] Nowakowski GS, Czuczman MS

[38] Ennishi D, Jiang A, Boyle M (2019). Double-hit gene expression signature defines a distinct subgroup of germinal center B-cell-like diffuse large B-cell lymphoma. J Clin Oncol.

[39] Sha C, Barrans S, Cucco F (2019). Molecular high-grade B-cell lymphoma: defining a poor-risk group that requires different approaches to therapy. J Clin Oncol.

[40] Cucco F, Barrans S, Sha C (2020). Distinct genetic changes reveal evolutionary history and heterogeneous molecular grade of DLBCL with MYC/BCL2 double-hit. Leukemia.

[41] Felsher DW, Bishop JM (1999). Reversible tumorigenesis by MYC in hematopoietic lineages. Mol Cell.

[42] Jain M, Arvanitis C, Chu K (2002). Sustained loss of a neoplastic phenotype by brief inactivation of MYC. Science.

[43] Pelengaris S, Khan M, Evan GI (2002). Suppression of Myc-induced apoptosis in beta cells exposes multiple oncogenic properties of Myc and triggers carcinogenic progression. Cell.

[44] Marinkovic D, Marinkovic T, Mahr B, Hess J, Wirth T (2004). Reversible lymphomagenesis in conditionally c-MYC expressing mice. Int J Cancer.

[45] Soucek L, Whitfield J, Martins CP (2008). Modelling Myc inhibition as a cancer therapy. Nature.

[46] Soucek L, Whitfield JR, Sodir NM (2013). Inhibition of Myc family proteins eradicates KRas-driven lung cancer in mice. Genes Dev.

[47] Mita MM, Mita AC (2020). Bromodomain inhibitors a decade later: a promise unfulfilled?. Br J Cancer.

[48] Chen H, Liu Z, Zheng L, Wang R, Shi L (2022). BET inhibitors: an updated patent review (2018-2021). Expert Opin Ther Pat.

[49] Johnson-Farley N, Veliz J, Bhagavathi S, Bertino JR (2015). ABT-199, a BH3 mimetic that specifically targets Bcl-2, enhances the antitumor activity of chemotherapy, bortezomib and JQ1 in “double hit” lymphoma cells. Leuk Lymphoma.

[50] Cinar M, Rosenfelt F, Rokhsar S (2015). Concurrent inhibition of MYC and BCL2 is a potentially effective treatment strategy for double hit and triple hit B-cell lymphomas. Leuk Res.

[51] Esteve-Arenys A, Valero JG, Chamorro-Jorganes A (2018). The BET bromodomain inhibitor CPI203 overcomes resistance to ABT-199 (venetoclax) by downregulation of BFL-1/A1 in in vitro and in vivo models of MYC^+^/BCL2^+^ double hit lymphoma. Oncogene.

[52] Shorstova T, Foulkes WD, Witcher M (2021). Achieving clinical success with BET inhibitors as anti-cancer agents. Br J Cancer.

[53] Li W, Gupta SK, Han W (2019). Targeting MYC activity in double-hit lymphoma with MYC and BCL2 and/or BCL6 rearrangements with epigenetic bromodomain inhibitors. J Hematol Oncol.

[54] Dickinson M, Briones J, Herrera AF (2021). Phase 1b study of the BET protein inhibitor RO6870810 with venetoclax and rituximab in patients with diffuse large B-cell lymphoma. Blood Adv.

[55] Winkler R, Magdefrau AS, Piskor EM (2022). Targeting the MYC interaction network in B-cell lymphoma via histone deacetylase 6 inhibition. Oncogene.

[56] Ho TCS, Chan AHY, Ganesan A (2020). Thirty years of HDAC inhibitors: 2020 insight and hindsight. J Med Chem.

[57] Guan XW, Wang HQ, Ban WW (2020). Novel HDAC inhibitor Chidamide synergizes with Rituximab to inhibit diffuse large B-cell lymphoma tumour growth by upregulating CD20. Cell Death Dis.

[58] Kang J, Zhang Y, Ding S (2021). Modified conditioning regimen with chidamide and high-dose rituximab for triple-hit lymphoma. J Cell Mol Med.

[59] Luo C, Yu T, Young KH, Yu L (2022). HDAC inhibitor chidamide synergizes with venetoclax to inhibit the growth of diffuse large B-cell lymphoma via down-regulation of MYC, BCL2, and TP53 expression. J Zhejiang Univ Sci B.

[60] Farrell AS, Sears RC (2014). MYC degradation. Cold Spring Harb Perspect Med.

[61] Kim SJ, Kim UJ, Yoo HY, Choi YJ, Kang KW (2020). Anti-cancer effects of CKD-581, a potent histone deacetylase inhibitor against diffuse large B-cell lymphoma. Int J Mol Sci.

[62] Li H, Cui R, Ji M, Jin SY (2021). CUDC-101 enhances the chemosensitivity of gemcitabine-treated lymphoma cells. Leuk Res.

[63] Zhang K, Huang L, Lai F (2022). Bioevaluation of a dual PI3K/HDAC inhibitor for the treatment of diffuse large B-cell lymphoma. Bioorg Med Chem Lett.

[64] Zhang MC, Fang Y, Wang L (2020). Clinical efficacy and molecular biomarkers in a phase II study of tucidinostat plus R-CHOP in elderly patients with newly diagnosed diffuse large B-cell lymphoma. Clin Epigenetics.

[65] Landsburg DJ, Barta SK, Ramchandren R (2021). Fimepinostat (CUDC-907) in patients with relapsed/refractory diffuse large B cell and high-grade B-cell lymphoma: report of a phase 2 trial and exploratory biomarker analyses. Br J Haematol.

[66] Sonawane YA, Taylor MA, Napoleon JV, Rana S, Contreras JI, Natarajan A (2016). Cyclin dependent kinase 9 inhibitors for cancer therapy. J Med Chem.

[67] Diamond JR, Boni V, Lim E (2022). First-in-human dose-escalation study of cyclin-dependent kinase 9 inhibitor VIP152 in patients with advanced malignancies shows early signs of clinical efficacy. Clin Cancer Res.

[68] Moreno V, Cordoba R, Morillo D (2021). Safety and efficacy of VIP152, a CDK9 inhibitor, in patients with double-hit lymphoma (DHL). J Clin Oncol.

[69] Frigault MM, Wong H, Garban H (2021). VIP152, a selective CDK9 inhibitor, induces complete regression in a high-grade B-cell lymphoma (HGBL) model and depletion of short-lived oncogenic driver transcripts, MYC and MCL1, with a once weekly schedule. Blood.

[70] Ball B, Borthakur G, Stein AS, Chan K, Thai DL, Stein E (2022). Safety and efficacy of casein kinase 1α and cyclin dependent kinase 7/9 inhibition in patients with relapsed or refractory AML: a first-in-human study of BTX-A51. J Clin Oncol.

[71] Ball BJ, Stein AS, Borthakur G (2020). Trial in progress: a phase I trial of BTX-A51 in patients with relapsed or refractory AML or high-risk MDS. Blood.

[72] Richters A, Doyle SK, Freeman DB (2021). Modulating androgen receptor-driven transcription in prostate cancer with selective CDK9 inhibitors. Cell Chem Biol.

[73] Day MA, Saffran DC, Hood T (2022). CDK9 inhibition via KB-0742 is a potential strategy to treat transcriptionally addicted cancers. Cancer Res.

[74] Mishra R, Patel H, Alanazi S, Kilroy MK, Garrett JT (2021). PI3K inhibitors in cancer: clinical implications and adverse effects. Int J Mol Sci.

[75] Chen L, Ouyang J, Wienand K (2020). CXCR4 upregulation is an indicator of sensitivity to B-cell receptor/PI3K blockade and a potential resistance mechanism in B-cell receptor-dependent diffuse large B-cell lymphomas. Haematologica.

[76] Grande BM, Gerhard DS, Jiang A (2019). Genome-wide discovery of somatic coding and noncoding mutations in pediatric endemic and sporadic Burkitt lymphoma. Blood.

[77] Profitos-Peleja N, Santos JC, Marin-Niebla A, Roue G, Ribeiro ML (2022). Regulation of B-cell receptor signaling and its therapeutic relevance in aggressive B-cell lymphomas. Cancers.

[78] Stengel S, Petrie KR, Sbirkov Y (2022). Suppression of MYC by PI3K/AKT/mTOR pathway inhibition in combination with all-trans retinoic acid treatment for therapeutic gain in acute myeloid leukaemia. Br J Haematol.

[79] Zhu J, Blenis J, Yuan J (2008). Activation of PI3K/Akt and MAPK pathways regulates Myc-mediated transcription by phosphorylating and promoting the degradation of Mad1. Proc Natl Acad Sci USA.

[80] Fan L, Wang C, Zhao L (2020). SHC014748M, a novel selective inhi-bitor of PI3Kδ, demonstrates promising preclinical antitumor activity in B cell lymphomas and chronic lymphocytic leukemia. Neoplasia.

[81] Zuo W-Q, Hu R, Wang W-L (2020). Identification of a potent and selective phosphatidylinositol 3-kinase δ inhibitor for the treatment of non-Hodgkin’s lymphoma. Bioorg Chem.

[82] Chen ZQ, Cao ZR, Wang Y (2022). Repressing MYC by targeting BET synergizes with selective inhibition of PI3Kalpha against B cell lymphoma. Cancer Lett.

[83] Zhang X, Duan YT, Wang Y (2022). SAF-248, a novel PI3Kdelta-selective inhibitor, potently suppresses the growth of diffuse large B-cell lymphoma. Acta Pharmacol Sin.

[84] Nastoupil LJ, Neelapu SS, Davis RE (2021). Preclinical and phase I studies of KA2237, a selective and potent inhibitor of PI3K β/δ in relapsed refractory B cell lymphoma. Leuk Lymphoma.

[85] Lee BR, Wang S, Son Mk (2020). Abstract 655: BR101801: a first-in-class triple-inhibitor of PI3K gamma/delta and DNA-PK targeting non-Hodgkin’s lymphoma. Cancer Res.

[86] Kim TM, Yoon DH, Mattour AH (2021). A phase 1 dose escalation study of dual PI3K and DNA PK inhibitor, BR101801 in adult patients with advanced hematologic malignancies. Blood.

[87] Shin N, Stubbs M, Koblish H (2020). Parsaclisib Is a next-generation phosphoinositide 3-kinase δ inhibitor with reduced hepatotoxicity and potent antitumor and immunomodulatory activities in models of B-cell malignancy. J Pharmacol Exp Ther.

[88] Wang Y, Laplant B, King RL (2021). Parsaclisib in combination with R-CHOP for patients with newly diagnosed diffuse large B-cell lymphoma: preliminary results of a phase 1/1b study. Blood.

[89] Moyo TK, Wilson CS, Moore DJ, Eischen CM (2017). Myc enhances B-cell receptor signaling in precancerous B cells and confers resistance to Btk inhibition. Oncogene.

[90] Ribeiro ML, Reyes-Garau D, Vinyoles M (2021). Antitumor activity of the novel BTK inhibitor TG-1701 is associated with disruption of ikaros signaling in patients with B-cell non-hodgkin lymphoma. Clin Cancer Res.

[91] Burke GAA, Beishuizen A, Bhojwani D (2020). Ibrutinib plus CIT for R/R mature B-NHL in children (SPARKLE trial): initial safety, pharmacokinetics, and efficacy. Leukemia.

[92] Burke GAA, Vinti L, Kabickova E (2022). Ibrutinib plus RICE/RVICI for R/R mature B-NHL in children/young adults: SPARKLE trial. Blood Adv.

[93] Yang H, Xiang B, Song Y (2022). Zanubrutinib monotherapy for relapsed or refractory non-germinal center diffuse large B-cell lymphoma. Blood Adv.

[94] Barr PM, Smith SD, Roschewski MJ (2022). Phase 1/2 study of acalabrutinib and the PI3K delta inhibitor ACP-319 in relapsed/refractory B-cell non-hodgkin lymphoma. Leuk Lymphoma.

[95] Beaulieu ME, Castillo F, Soucek L (2020). Structural and biophysical insights into the function of the intrinsically disordered Myc oncoprotein. Cells.

[96] Gomez-Curet I, Perkins RS, Bennett R, Feidler KL, Dunn SP, Krueger LJ (2006). c-Myc inhibition negatively impacts lymphoma growth. J Pediatr Surg.

[97] Clausen DM, Guo J, Parise RA (2010). In vitro cytotoxicity and in vivo efficacy, pharmacokinetics, and metabolism of 10074-G5, a novel small-molecule inhibitor of c-Myc/max dimerization. J Pharmacol Exp Ther.

[98] Choi SH, Mahankali M, Lee SJ (2017). Targeted disruption of Myc-max oncoprotein complex by a small molecule. ACS Chem Biol.

[99] Pighi C, Cheong TC, Compagno M (2021). Frequent mutations of FBXO11 highlight BCL6 as a therapeutic target in Burkitt lymphoma. Blood Adv.

[100] Li X-Y, Wu J-C, Liu P (2023). Inhibition of USP1 reverses the chemotherapy resistance through destabilization of MAX in the relapsed/refractory B-cell lymphoma. Leukemia.

[101] Sun Y, Yang L, Hao X (2019). Phase I dose-escalation study of chiauranib, a novel angiogenic, mitotic, and chronic inflammation inhibitor, in patients with advanced solid tumors. J Hematol Oncol.

[102] Deng M, Shi Y, Chen K (2018). CS2164 exerts an antitumor effect against human non-Hodgkin’s lymphomas in vitro and in vivo. Exp Cell Res.

[103] Yuan D, Li G, Yu L (2021). CS2164 and venetoclax show synergistic antitumoral activities in high grade B-cell lymphomas with MYC and BCL2 rearrangements. Front Oncol.

[104] Thompson PA, Eam B, Young NP (2021). Targeting oncogene mRNA translation in B-cell malignancies with eFT226, a potent and selective inhibitor of eIF4A. Mol Cancer Ther.

[105] Moreau P, Richardson PG, Cavo M (2012). Proteasome inhibitors in multiple myeloma: 10 years later. Blood.

[106] Ravi D, Beheshti A, Abermil N (2016). Proteasomal inhibition by ixazomib induces CHK1 and MYC-dependent cell death in T-cell and hodgkin lymphoma. Cancer Res.

[107] Graf SA, Lynch RC, Gooley TA (2020). Ixazomib-rituximab in untreated indolent B-NHL: an effective, very low toxicity regimen. Blood.

[108] Galvez C, Karmali R, Hamadani M (2020). A phase I-II trial of DA-EPOCH-R plus ixazomib as frontline therapy for patients with MYC-Aberrant lymphoid malignancies: the daciphor regimen. Blood.

[109] Davies AJ, Stanton L, Caddy J (2022). Five-year survival results from the remodl-B trial (ISRCTN 51837425) show improved outcomes in diffuse large B-cell lymphoma molecular subgroups from the addition of bortezomib to R-CHOP chemoimmunotherapy. Blood.

[110] Davies A, Cummin TE, Barrans S (2019). Gene-expression profiling of bortezomib added to standard chemoimmunotherapy for diffuse large B-cell lymphoma (REMoDL-B): an open-label, randomised, phase 3 trial. Lancet Oncol.

[111] Strati P, Nastoupil LJ, Davis RE (2020). A phase 1 trial of alisertib and romidepsin for relapsed/refractory aggressive B-cell and T-cell lymphomas. Haematologica.

[112] Gavory G, Ghandi M, d’Alessandro A-C (2022). Abstract 3929: identification of MRT-2359 a potent, selective and orally bioavailable GSPT1-directed molecular glue degrader (MGD) for the treatment of cancers with Myc-induced translational addiction. Cancer Res.

[113] Sesques P, Johnson NA (2017). Approach to the diagnosis and treatment of high-grade B-cell lymphomas with MYC and BCL2 and/or BCL6 rearrangements. Blood.

[114] Rosenwald A, Bens S, Advani R (2019). Prognostic significance of MYC rearrangement and translocation partner in diffuse large B-cell lymphoma: a study by the lunenburg lymphoma biomarker consortium. J Clin Oncol.

[115] Ott G, Rosenwald A, Campo E (2013). Understanding MYC-driven aggressive B-cell lymphomas: pathogenesis and classification. Blood.

[116] Petrich AM, Nabhan C, Smith SM (2014). MYC-associated and double-hit lymphomas: a review of pathobiology, prognosis, and therapeutic approaches. Cancer.

